# A *Drosophila* model of HPV16-induced cancer reveals conserved disease mechanism

**DOI:** 10.1371/journal.pone.0278058

**Published:** 2022-12-12

**Authors:** Lydia Hashemi, McKenzi E. Ormsbee, Prashant J. Patel, Jacquelyn A. Nielson, Joseph Ahlander, Mojgan Padash Barmchi

**Affiliations:** 1 Department of Biology, University of Oklahoma, Norman, OK, United States of America; 2 Stead Family Department of Pediatrics, University of Iowa, Iowa City, IA, United States of America; 3 Department of Natural Sciences, Northeastern State University, Broken Arrow, OK, United States of America; University of Dayton, UNITED STATES

## Abstract

High-risk human papillomaviruses (HR-HPVs) cause almost all cervical cancers and a significant number of vaginal, vulvar, penile, anal, and oropharyngeal cancers. HPV16 and 18 are the most prevalent types among HR-HPVs and together cause more than 70% of all cervical cancers. Low vaccination rate and lack of molecularly-targeted therapeutics for primary therapy have led to a slow reduction in cervical cancer incidence and high mortality rate. Hence, creating new models of HPV-induced cancer that can facilitate understanding of the disease mechanism and identification of key cellular targets of HPV oncogenes are important for development of new interventions. Here in this study, we used the tissue-specific expression technique, Gal4-UAS, to establish the first *Drosophila* model of HPV16-induced cancer. Using this technique, we expressed HPV16 oncogenes E5, E6, E7 and the human E3 ligase (hUBE3A) specifically in the epithelia of *Drosophila* eye, which allows simple phenotype scoring without affecting the viability of the organism. We found that, as in human cells, hUBE3A is essential for cellular abnormalities caused by HPV16 oncogenes in flies. Several proteins targeted for degradation by HPV16 oncoproteins in human cells were also reduced in the *Drosophila* epithelial cells. Cell polarity and adhesion were compromised, resulting in impaired epithelial integrity. Cells did not differentiate to the specific cell types of ommatidia, but instead were transformed into neuron-like cells. These cells extended axon-like structures to connect to each other and exhibited malignant behavior, migrating away to distant sites. Our findings suggest that given the high conservation of genes and signaling pathways between humans and flies, the *Drosophila* model of HPV16- induced cancer could serve as an excellent model for understanding the disease mechanism and discovery of novel molecularly-targeted therapeutics.

## Introduction

High-risk human papillomaviruses (HR-HPVs) are the leading cause of cervical, vaginal, vulvar, penile, anal, and a significant number of oropharyngeal cancers. Annually, roughly 750,000 of HR-HPV-induced cancer cases are identified, of which the majority are caused by HPV16 and HPV18 [1–3]. HPV16 appears to be the most prevalent carcinogenic HR-HPV subtype accounting for more than 80% of the HPV-induced head and neck squamous carcinomas and more than 50% of cervical cancers [4–7]. Despite the availability of HPV vaccines, a reduction in the rate of HPV-associated cancers has been slow due to a number of factors, including the low rate of vaccine uptake, the long delay between initial infection and the development of malignancy, and the fact that the current vaccines are prophylactic rather than therapeutic and thus cannot treat pre-existing infections. In more than 90% of individuals infected with HPV, the immune system eliminates the infection within 12–24 months. However, in a small percentage of infected cases (5–10%), the host immune system does not clear the infection and this can lead to the development of cancer. The underlying mechanism is not well-understood, however, it appears that the persistent HPV infection is necessary for cancer development and progression towards malignancy. Persistent HPV infection leads to disruption of cellular homeostasis through cell cycle deregulation, apoptosis evasion, and suppression of the host immune response. This in turn results in increased cell proliferation, genomic instability, immortalization, cell transformation, and progression towards malignancy. The main drivers of these destructive actions are the three HPV oncogenes, E5, E6, and E7 [8–10].

E5, though a weak transforming protein *in vitro*, provides support for infection and transformation of cells. It is expressed during the early phases of the viral life cycle and stimulates cell proliferation and transformation by enhancing the activation of the epidermal growth factor receptor (EGFR) pathway, downregulation of the anti-proliferative keratinocyte growth factor receptor (KGFR), activation of MAPK signaling, and inhibition of cell cycle regulators [11–16]. *In vivo* studies showed that E5 enhances the oncogenic abilities of E6 and E7 in cellular transformation and tumor formation [17]. Furthermore, E5 protects infected cells from apoptosis through several mechanisms including proteasomal-mediated degradation of pro-apoptotic Bcl-2 family member Bax and by reducing the expression of necrosis factor ligand (FasL) receptor and altering the formation of the Death-Inducing Signaling Complex (DISC) [18, 19].

E6 promotes tumor formation and cell immortalization by targeting specific proteins and dysregulating several cellular signaling pathways. These proteins include the tumor suppressor protein p53, the pro-apoptotic protein BAK, and several key regulators of cell polarity and junctions including PDZ domain proteins Dlg, Scribble, and the Magi family of proteins [20–27]. E6 interacts with these proteins by either binding to them to inhibit their function or by mediating their degradation, decreasing the levels of target proteins in transformed cells. There are two important domains of E6 that allow it to interact with its targets. The first domain is the PDZ binding domain, which allows E6 to attach to its PDZ targets [28]. The second domain of E6 is its α-helix binding domain. Here, E6 binds to the human ubiquitin protein-ligase E6AP/UBE3A to induce the degradation of target proteins through the ubiquitin-proteasome degradation pathway. Targeting the PDZ domain protein is important for HPV-induced tumor formation, as E6 defective in p53 degradation can still immortalize cells, whereas E6 deficient in PDZ binding fails to induce hyperplasia [29, 30]. E6 also dysregulates several signaling pathways including Wnt, JAK/STAT, Hippo, Notch, and PI3K/AKT/mTOR to promote cell growth, survival, and proliferation [10].

E7, known as the major transforming oncoprotein of HPV, promotes increased cell proliferation by dysregulating the G1/S transition. This is achieved by either binding retinoblastoma (Rb) and releasing it from the E2F transcription factor, or by proteasomal-mediated degradation of Rb [31, 32]. E7 also delays cell differentiation through targeting PTPN14 for proteasomal-mediated degradation [33–35]. While E6 and E7 can individually immortalize the cells, studies have indicated that they cooperate in tumorigenesis with E7 being involved in the early stages of tumor formation whilst E6 accelerates progression towards malignancy [36, 37].

While much is known about HPV oncogenes and their targets, the mechanisms underlying how the disruption of cellular homeostasis leads to cancer remain to be elucidated. The majority of what is known about HPV oncogenes and targets is a result of *in vitro* and transgenic mice studies, yet these tools have yet to determine the disease mechanism or identify effective therapeutic treatments. A *Drosophila melanogaster* can serve as a well-rounded model for HPV16-induced cancer, representing the whole organism aspect of the disease that *in vitro* models lack, and providing a model that is more efficient and cost-effective than transgenic mice models for genetic screening and high-throughput compound and drug screening [38–41]. *Drosophila* has been used as a powerful genetic model organism for studying human diseases, as up to 75% of disease-related genes in humans have functional *Drosophila* orthologs [42–44]. Similarly fruit flies have played important role in understanding the mechanisms underlying the interactions between pathogens including viruses with their host, uncovering the molecular mechanism of tissue regeneration, and identifying new therapeutics derived from plants and other organisms [45–47]. We have previously established a *Drosophila* model of HPV18-induced cell deregulation and successfully used it to identify important players in inducing the cellular abnormalities caused by HPV18 oncogenes [48, 49]. Therefore, a *Drosophila* model of HPV16-induced cancer will be a powerful complement to existing *in vitro* and transgenic mice models in the study of cancerous pathways driven by HPV16 oncogenes.

Here in this study, we used the tissue-specific expression system known as Gal4/UAS to express three main oncoproteins of HPV16 E5, E6, and E7, plus the human UBE3A, exclusively in the *Drosophila* eye epithelia. The expression of oncogenes resulted in lethality at the pupal stage. We found that, as in human cells, hUBE3A is essential for cellular abnormalities caused by HPV16 oncogenes in flies. Additionally, we discovered that several proteins targeted for degradation by HPV16 oncoproteins in human cells are also targeted in the *Drosophila* epithelial cells. Similarly, cell polarity and adhesion were compromised, resulting in impaired epithelial integrity. Cells did not differentiate to specific cell types of ommatidia, but instead were transformed into neuron-like cells. These cells extended axon-like structures to connect to each other and exhibited malignant behavior, migrating away from the place of origin to distant sites. Our findings suggest that given the high conservation of genes and signaling pathways between humans and flies, the *Drosophila* model of HPV16- induced cancer could serve as an excellent model for the understanding of disease mechanisms, as well as for identification of novel therapeutic targets for the treatment of cervical cancer and perhaps all HPV-induced cancers, nationwide and globally.

## Materials and methods

### Fly strains

The following fly stocks were used: UAS-HPV16 E5, E6, E7 (generated in this study), UAS-hUBE3A, GMR-Gal4, UAS-p35, UAS-mCD8RFP, and CS were from Bloomington *Drosophila* stock center.

### Generation of UAS-HPV16 E5, E6, E7 transgenic strain

The construct was designed to be multicistronic and encode three separate proteins from the same transcript by use of T2A polyprotein cleavage sequences [50]. Three proteins are separated by a ribosomal-skip mechanism in which a peptide bond fails to form between the Glycine and Proline residues within the 2A peptide sequence without translation being halted [51]. This method allows the production of several transgenes without the issue of disproportionate transgene expression levels [52, 53]. E7 and E6 have a C-terminal T2A epitope tag, and E5 has an N-terminal HA epitope tag. HPV coding sequences were codon optimized for expression in *Drosophila* and synthesized by Biomatik. PCR-derived gene fragments were assembled with InFusion cloning (Takara Bio) using the pJFRC7 plasmid as a backbone expression vector [54]. The assembled construct was isolated from a single clone and verified by sequencing. Transgenic flies were generated by phiC31 integrase insertion into the attP2 site on chromosome 3 [55] by Duke University Model Systems Genomics.

### Immunohistochemistry

For immunolabeling either 3^rd^ instar larval eye imaginal discs or pupal eyes, 40–42 hours after puparium formation (APF), were dissected in PBS and fixed in 4% formaldehyde. Fixed tissues were washed three times in PBS solution containing 0.1% Triton-X-100 and blocked in 5% normal goat serum for 1 h before incubation with primary antibodies. The primary antibodies used were: rat anti-DE-cadherin (DCAD2) 1:50, mouse anti-Dlg (4F3) 1:100, mouse anti-Futsch (22C10) 1:100, mouse anti-Arm (N2 7A1) 1:50, and mouse anti-dE2F1 (Hao4) 1:20 from Developmental Studies Hybridoma Bank, rabbit anti-Magi 1:200 [56], rabbit anti-cleaved caspase 3 (9578, Cell Signaling), rabbit anti-aPKC zeta C-20 1:1000 (SC-216, Santa Cruz Biotechnology), mouse anti-RBF1 1:20 (a generous gift from Dr. Maxim Frolov). The primary antibodies were detected using the following secondary antibodies: goat anti-rabbit Alexa 488, goat anti-mouse Alexa 488, and goat anti-rat Alexa 488 which were all from Invitrogen. The images were generated using a Leica SP8 confocal microscope and processed in ImageJ. The figures were generated using Adobe Photoshop. For quantification of protein levels, the fluorescence intensity was measured using ImageJ in both the transgenes expressing and non-expressing regions. To measure the fluorescence intensity, the maximal intensity projection images were created based on the z-series of images. Default thresholding and "analyze particles" with size limitation over 2000 um^2^ was applied to select area in transgenes expressing region versus the non-expressing region that was marked using the "inverse selection" function. The intensity of fluorescent probes of both regions was measured and the data were then transferred to Excel for further analysis and plot creation. The average percentage of intensity changes between the transgenes expressing and non-expressing regions were plotted. For each experiment, at least six eye discs were analyzed and a paired t-test was used for statistical analysis.

## Results

### Expression of HPV16 E5, E6, and E7 oncogenes leads to mortality in a human UBE3A-dependent manner

**Fig 1:**
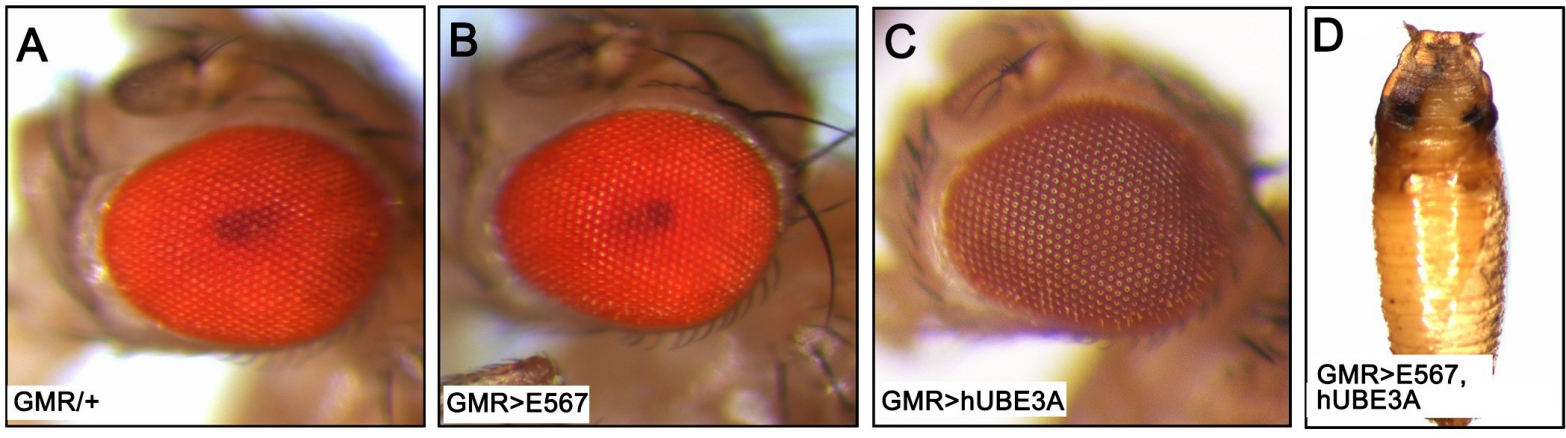
Expression of HPV16 E5, E6, and E7 oncogenes leads to mortality in a human UBE3A-dependent manner. Transgenes were expressed using the GMR-Gal4 in the eye discs **(A-D)**. The wild type **(A)** and expression of HPV16 E5, E6, E7 alone **(B)** or hUBE3A **(C)** showed no phenotypic defects in the adult fly eye. Flies co-expressing E5, E6, E7 and hUBE3A **(D)** did not develop beyond the pupal stage.

The simple epithelia of the *Drosophila* eye provide a useful model for studying epithelial-derived human cancer as genetic manipulations in the eye epithelia typically do not affect the survival of the animal. Furthermore, cellular dysfunction in the *Drosophila* compound eye manifests as a rough eye phenotype that can easily be observed. Hence, we expressed HPV16 oncogenes E5, E6, and E7 exclusively in the eye epithelia using the Gal4-UAS binary expression system. This system takes advantage of the Gal4 transcription activator, whose expression is under the control of the eye-specific gene promoter GMR, to bind to the UAS sequence and activate the expression of whatever gene follows the sequence [57]. We found that the expression of these oncogenes alone did not have any effect on the morphology of the eye. The eyes exhibited morphology that was similar to the morphology of control eyes where the GMR-Gal4 was only expressed (Fig 1A and 1B). This result indicated that firstly, E6 requires the activity of E3 ubiquitin ligase (UBE3A) to exert its effect and that it is unable to interact with *Drosophila* UBE3A as *Drosophila* UBE3A lacks the E6-binding motif [49]. Secondly, as it was shown previously, cooperation of E6 and E7 is necessary for cell transformation [37]. In support of this notion, we found that when HPV16 oncogenes were co-expressed with human UBE3A (hUBE3A) [58] the animals did not develop beyond the pupal stage, resulting in lethality (Fig 1D). This result was in contrast to expression of hUBE3A alone, which did not induce any eye abnormalities (Fig 1C). Altogether, these results indicated that E5, E6, E7 are not sufficient to cause altered morphology and that E6 requires the assistance of hUBE3A for its destructive actions.

### HPV16 E5, E6, E7 oncoproteins disrupt epithelial integrity

**Fig 2:**
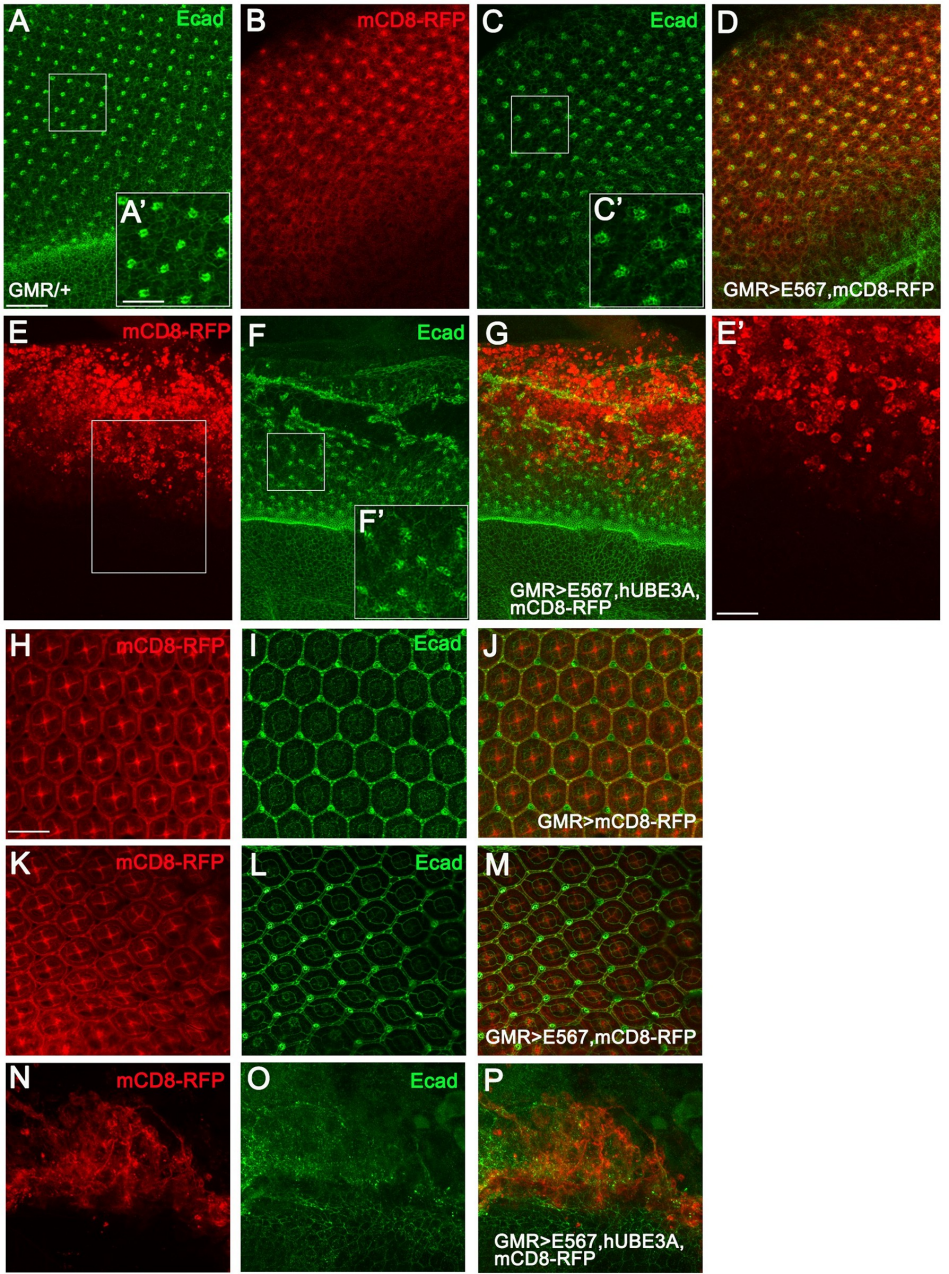
HPV16 E5, E6, E7 oncoproteins disrupt epithelial integrity. Transgenes were expressed in the third instar larval eye discs (**A-E’)** and in pupal eyes 42 hours after pupation **(H-P),** using GMR-Gal4. Tissues were immunolabelled for adherens junction protein, E-cad and the expression of mCD8RFP was used to mark the area of transgenes expression. **(A)** GMR-Gal4-expressing eye disc showing an intact epithelium with proper localization of E-cad **(A’)** and a sequential pattern of differentiated ommatidia. **(B-D)** Co-expression of HPV16 E5, E6, E7 and the membrane marker, mCD8RFP showing no cellular abnormalities as indicated by proper localization of E-cad and ommatidia formation **(C, C’)**. **(E-G)** Eye disc co-expressing HPV16 E5, E6, E7+hUBE3A+ mCD8RFP exhibited mislocalization or loss of E-cad **(F, F’)**, rounded cell morphology **(E, E’)**, and disrupted pattern of ommatidial formation **(F)**. **(H-J)** Pupal eye 42 hrs after pupation expressing GMR-Gal4 and mCD8RFP showing a fully differentiated eye with a stereotype pattern of hexagonal array of ommatidia and proper localization of E-cad **(I)**. **(K-M)** Pupal eye co-expressing HPV16 E5, E6, E7+ mCD8RFP showed no cellular abnormalities and eyes exhibited similar morphology and E-cad localization to the control eyes in H-J. **(N-P)** Pupal eye co-expressing HPV16 E5, E6, E7+hUBE3A+ mCD8RFP showed no ommatidia formation and instead the pupal eye was clusters of cells **(N)** that had lost cell-cell adhesion evident by lack of E-cad. **(O)**. Insets are digitally magnified 200%. Scale bar indicates 100 μm in all panels except insets in which scale bar represents 50 μm.

*Drosophila* eye epithelium has proven to be an excellent system for understanding the cellular and molecular mechanisms underlying cell transformation and cancer [59–61]. To assess the effect of E5, E6, E7, and hUBE3A co-expression on the epithelial integrity, the eye tissues from two developmental stages were examined. These developmental stages were the 3^rd^ instar larval and 42 hours after puparium formation, when the eye is fully developed. Immunolabelling with adherens junction protein, E-Cad, revealed that while expression of GMR-Gal4 or (E5, E6, E7) alone did not disrupt cell-cell adhesion, in neither of the two stages examined (Fig 2A–2D and 2H–2M), did co-expression of E5, E6, E7+ hUBE3A perturbed adherens junctions, resulting in loss of epithelial integrity (Fig 2E–2G and 2N–2P). Eye discs expressing either (E5, E6, E7) or GMR-Gal4 driver exhibited an intact epithelium with proper cell differentiation and ommatidia formation, which further developed to an array of hexagonal ommatidia arranged in a stereotypical pattern at 42 hours after pupation. However, eye discs expressing E5, E6, E7+hUBE3A did not maintain an intact epithelium. These eye discs showed loss of epithelial integrity, which was evident by rounded cell morphology, and loss of epithelial cells (Fig 2E). The defects were more severe at the pupal stage, as no ommatidia were detected and the E5, E6, E7+hUBE3A-expressing eye was found to be a cluster of cells that had lost adherens junctions. These observations suggested that HPV16 E5, E6, E7 in cooperation with hUBE3A disrupts the cell-cell attachments and cell differentiation, both of which are hallmarks of HPV-induced cancers.

### Expression of HPV16 E5, E6, E7 oncogenes in conjunction with hUBE3A induces apoptosis in larval eye disc and has no effect on cell proliferation

**Fig 3:**
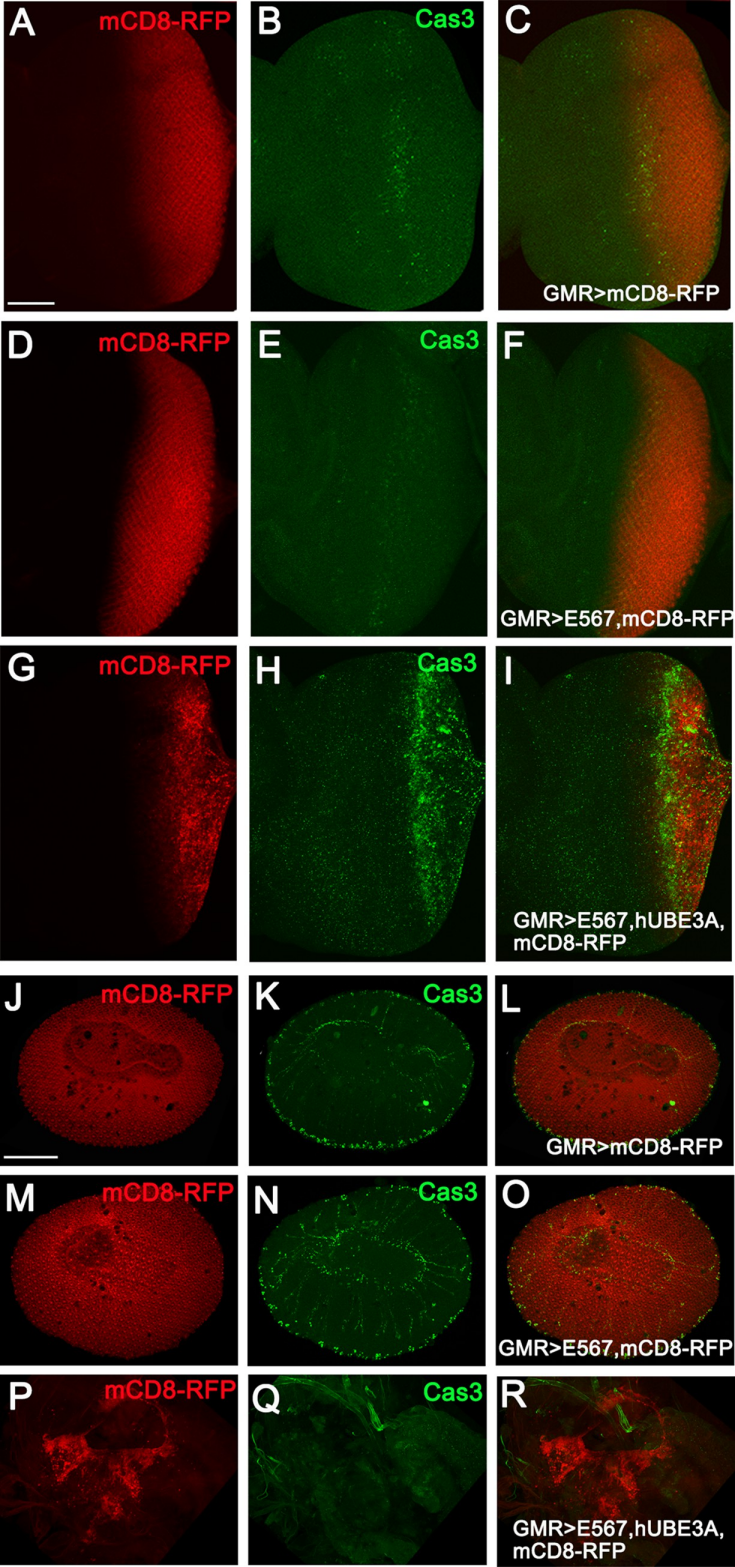
Expression of HPV16 E5, E6, E7 oncogenes in conjunction with hUBE3A induces apoptosis in larval eye disc. Transgenes were expressed in the third instar larval eye discs **(A-I)** and in pupal eyes 42 hours after pupation **(J-R)**, using GMR-Gal4. mCD8RFP was used to mark the area of transgenes expression and tissues were immunolabeled for apoptotic cell marker, Cleaved Caspase 3 (Cas3). **(A-C)** Eye disc expressing mCD8RFP exhibiting very few apoptotic cells present in the mCD8RFP-expressing region. **(D-F)** Eye disc co-expressing HPV16 E5, E6, E7 and mCD8RFP showing a similar result to control eye in A-C. **(G-I)** Co-expression of HPV16 E5, E6, E7+hUBE3A+ mCD8RFP resulted in significant increase in apoptosis **(H)**. However not all cells within the region underwent apoptosis **(I)**. **(J-L)** Pupal eye 42 hrs after pupation expressing mCD8RFP showing a ring of apoptotic ommatidia at the periphery of the eye. **(M-O)** Co-expression of HPV16 E5, E6, E7+ mCD8RFP resulted in similar result to the control eyes in J-L. **(P-R)** Pupal eye tissue co-expressing HPV16 E5, E6, E7+hUBE3A+ mCD8RFP showing lack of hexagonal array of ommatidia but instead clusters of non- apoptotic cells (P and Q). Scale bar in A is 50 μm and in J is 100 μm.

Loss of adherens junctions and epithelial integrity prompted us to determine whether cells expressing HPV oncogenes in conjunction with hUBE3A undergo programmed cell death. Immunolabeling with the apoptotic marker, cleaved caspase 3, revealed that many cells of the larval eye disc expressing E5, E6, E7+hUBE3A die due to apoptosis (Fig 3G–3I). This observation was in contrast to the eye discs expressing either (E5, E6, E7) or GMR-Gal4 driver alone as they exhibited only a few apoptotic cells (Fig 3A–3F). Since not all cells of larval eye discs expressing E5, E6, E7+hUBE3A underwent apoptosis, we asked whether the remainder of cells had resisted apoptosis and progressed to the pupal stages of the eye development. Interestingly, we did not detect any apoptotic cells among the E5, E6, E7+hUBE3A-expressing cells at 42 hours after pupation (Fig 3P–3R). This was in contrast to GMR-Gal4 or (E5, E6, E7)-expressing pupal eyes that showed a ring of apoptotic ommatidia in the periphery of the eye (Fig 3J–3O). This ring of apoptotic ommatidia is a natural mechanism that eliminates incomplete ommatidia from the eye periphery at the pupal stage [62]. These results suggested that HPV oncogenes in cooperation with hUBE3A, induce apoptosis in the eye disc epithelium. However, not all cells expressing E5, E6, E7+hUBE3A respond equally, as some epithelial cells resisted apoptosis, survived, and progressed to pupal stages.

**Fig 4:**
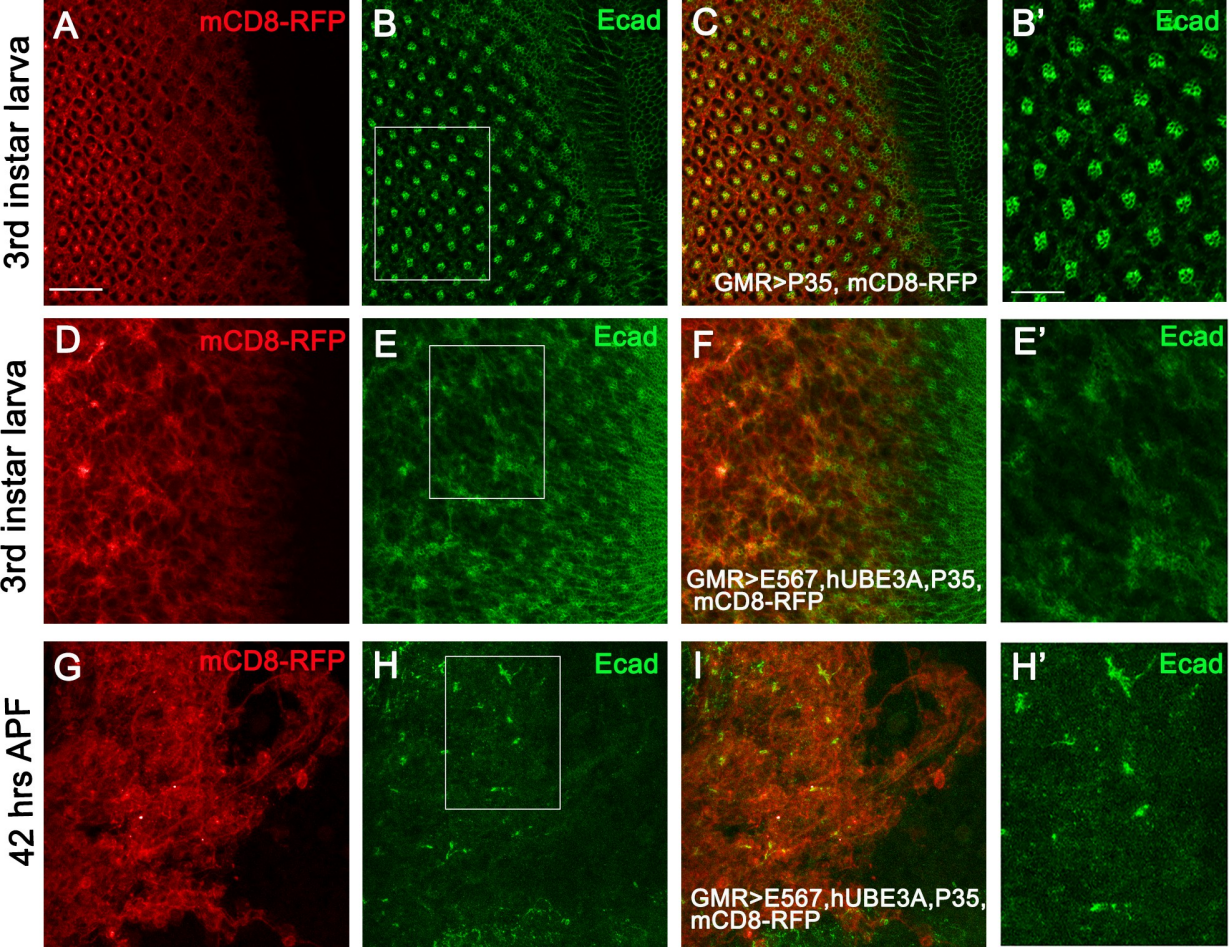
HPV16 E5, E6, E7+hUBE3A-induced loss of adherens junctions and impaired cell differentiation is independent of programmed cell death. Transgenes were expressed in the third instar larval eye discs **(A-E’)** and in pupal eyes 42 hours after pupation **(G-H’)**, using GMR-Gal4. mCD8RFP was used to mark the area of transgenes expression and tissues were immunolabeled for adherens junctions protein, E-cad. **(A-B’)** Eye disc co-expressing mCD8RFP and p35 showed an intact eye epithelium with proper sequential pattern of differentiated ommatidia **(B)** and adherens junctions **(B’)**. **(D-E’)** Eye disc co-expressing HPV16 E5, E6, E7+hUBE3A+ mCD8RFP +p35 exhibited loss of adherens junction protein **(E and E’)** and impaired ommatidial differentiation **(D)** despite p35-mediated absence of cell death. **(G-H’)** Pupal eye 42 hrs after pupation co-expressing HPV16 E5, E6, E7+hUBE3A+ mCD8RFP +p35 showed cluster of cells in which many cells lost adherens junctions whilst some remained attached to each other expressing the E-cad **(H, H’)**. Insets are digitally magnified 200%. Scale bars in A and B’ represent 100 μm and 50 μm, respectively.

In order to determine whether impaired cell differentiation and loss of epithelial integrity are due to apoptosis or an independent mechanism, we expressed a baculovirus protein p35 that blocks apoptosis, in conjunction with E5, E6, E7, and hUBE3A [63]. We found that when p35 was expressed alone in larval eye disc, an intact epithelium was present and cell differentiation occurred properly forming a sequential pattern of ommatidia (Fig 4A–4B’). However, eye discs co-expressing E5, E6, E7+hUBE3A+p35, despite the p35-mediated absence of cell delamination and apoptosis, exhibited impaired adherens junctions and cell differentiation (Fig 4D–4E’). These abnormalities were also present in the pupal eye as no ommatidia were detected. The eye tissue consisted of clusters of cells, many of which had lost adherens junctions, whilst others had established strong adhesions (Fig 4G–4H’). These results suggested that HPV16 E5, E6, E7+hUBE3A-induced loss of adherens junctions and impaired cell differentiation is independent of programmed cell death.

During the third instar larval development, a morphogenetic furrow appears near the posterior edge of the eye disc, which sweeps anteriorly in a progressive fashion. Cells posterior to the furrow are differentiated in a sequential pattern into ommatidia, whilst the cells anterior to the furrow are undifferentiated and randomly proliferate to expand the eye epithelium [64]. In order to determine whether the expression of HPV16 oncogenes alone, or in cooperation with hUBE3A, has any effect on cell proliferation, we stained the third instar larval eye discs with antibody against the phospho-histone 3 (pH3), which is a marker for mitotic cells [65]. Similar to the results of the previous experiments in this study, there are many similarities in the proliferation pattern of eye discs expressing GMR-Gal4 alone or the HPV oncogenes E5, E6, E7. Mitotic cells detected with pH3 were located anterior to the morphogenetic furrow (S1A–S1C and S1G Fig). A similar result was also obtained when HPV E5, E6, E7 were expressed in conjunction with hUBE3A (S1D–S1F Fig). These results suggested that the combined expression of HPV16 oncogenes and hUBE3A causes loss of epithelial integrity and apoptosis, with no effect on the mitotic cell division during larval eye disc patterning and development.

### HPV16 E5, E6, and E7 oncoproteins disrupt cellular polarity

**Fig 5:**
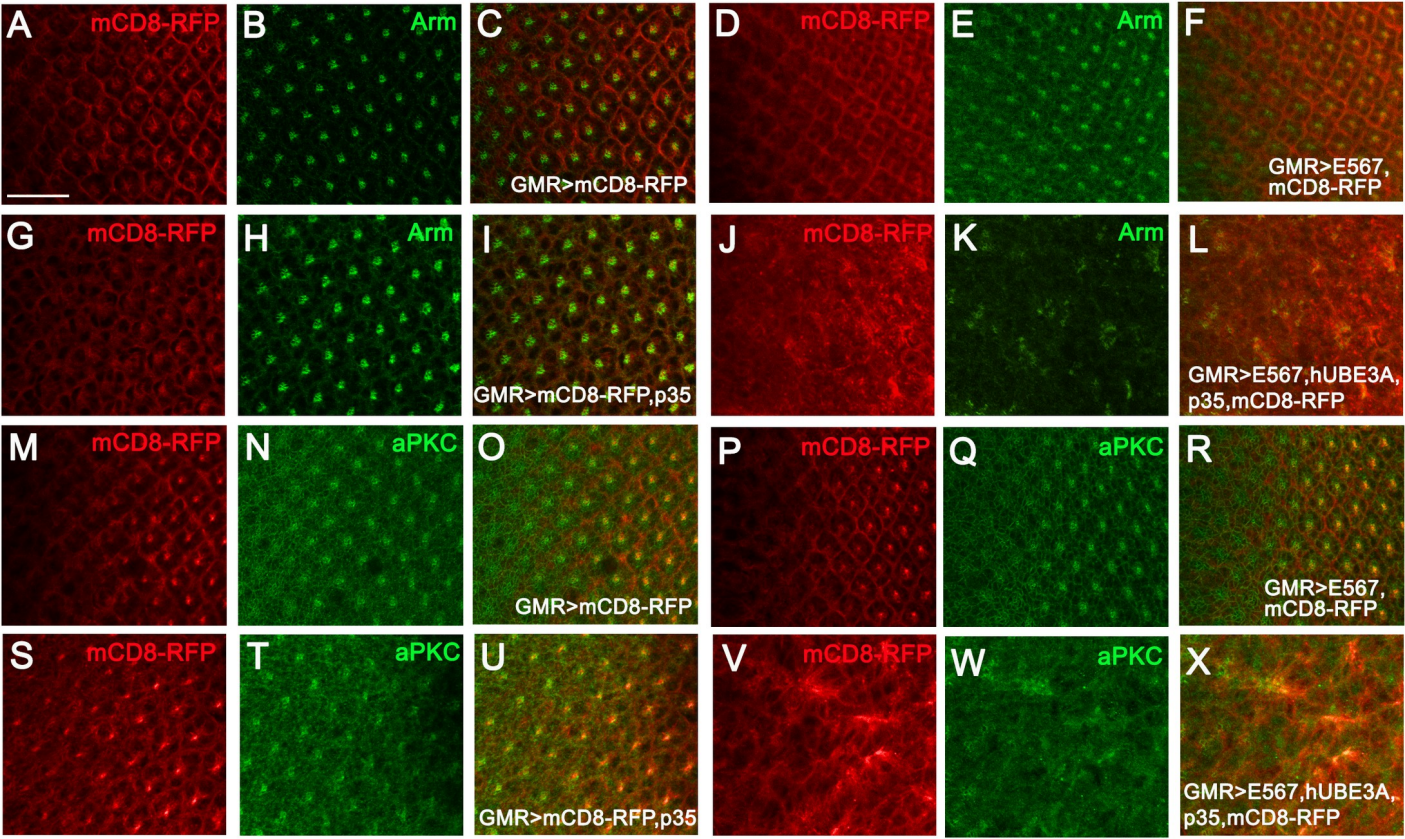
HPV16 E5, E6, and E7 oncoproteins disrupt cellular polarity. Transgenes were expressed using GMR-Gal4 in the third instar larval eye discs. mCD8RFP was used to mark the transgenes expression region and tissues were immunolabeled for polarity proteins, Arm and aPKC. **(A-C)** Eye disc expressing mCD8RFP exhibited an intact epithelium with a sequential pattern of ommatidia **(A)** and proper localization of Arm **(B)**. **(D-F)** Eye disc co-expressing E5, E6, E7+ mCD8RFP exhibited a similar result to control eye in A-C. **(G-I)** Eye disc co-expressing mCD8RFP+p35 showed an intact epithelium **(G)** with proper Arm localization **(H)** and ommatidia pattern to those in A-C and D-F. **(J-L)** Co-expression of E5, E6, E7+hUBE3A+ mCD8RFP +p35 resulted in impaired cell differentiation as no ommatidia had formed **(J)** and epithelial cell polarity was perturbed as Arm was either mislocalized or absent **(K)**. **(M-O)** Eye disc expressing mCD8RFP exhibited an intact epithelium with a sequential pattern of ommatidia **(M)** and proper localization of aPKC **(N)**. **(P-R)** Eye disc co-expressing E5, E6, E7+ mCD8RFP exhibited a similar result to control eye in M-O. **(S-U)** Eye disc co-expressing mCD8RFP+p35 showed an intact epithelium **(S)** with proper aPKC localization **(T)** and ommatidia pattern to those in M-O and P-R. **(V-X)** Co-expression of E5, E6, E7+hUBE3A+ mCD8RFP +p35 resulted in impaired cell differentiation as no ommatidia had formed **(V)** and epithelial cell polarity was perturbed as aPKC was absent **(W)**. Scale bar represents 100 μm.

As HPV16 oncogenes are known to disrupt human epithelial cell polarity to promote progression towards malignancy, we examined the E5, E6, E7 +hUBE3A+p35-expressing eye discs for the components of cell polarity control complexes, including beta-catenin (*Drosophila* Armadillo, Arm) and aPKC. These proteins are necessary for the polarity of eye epithelial cells before differentiation as well as for the polarity of the differentiated ommatidial cells. We found that expression of either GMR-Gal4, (E5, E6, E7), or p35 alone had no effect on the localization of aPKC or Arm, and a polarized epithelium was observed (Fig 5A–5I and 5M–5U). However, the epithelium of eye discs co-expressing E5, E6, E7 +hUBE3A+p35 had lost Arm and aPKC indicating loss of cell polarity (Fig 5J–5L and 5V–5X). These results are consistent with previous studies indicating a role for HPV oncogenes in perturbing cell polarity [66–68] and suggested that HPV16 E5, E6, E7-induced loss of cell polarity requires the activity of hUBE3A and is independent of apoptosis.

### HPV16 E5, E6, E7 reduce the levels of Retinoblastoma and PDZ domain proteins Magi and Dlg

**Fig 6:**
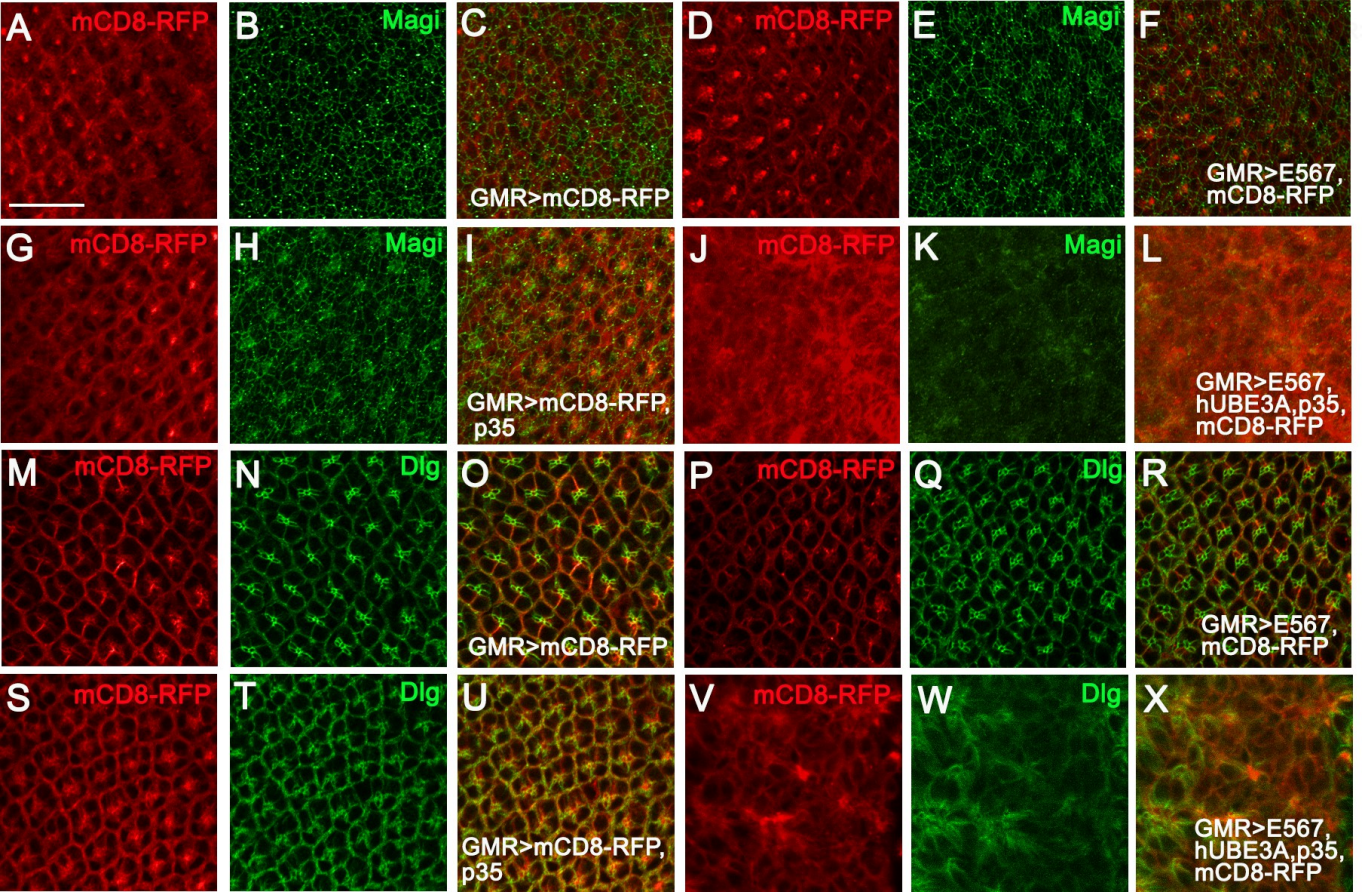
HPV16 E5, E6, E7 in conjunction with hUBE3A promote the degradation of PDZ domain proteins Magi and Dlg. Transgenes were expressed using the GMR-Gal4 in the third instar larval eye discs. mCD8RFP was used to mark the transgenes expression region and tissues were immunolabeled for PDZ domain proteins, Magi and Dlg. **(A-C)** Expression of mCD8RFP had no effect on Magi **(B)**. **(D-F)** Co-expression of mCD8RFP +E5, E6, E7 had no effect on Magi **(E)**. **(G-I)** Co-expression of mCD8RFP+p35 did not affect Magi level and localization **(H)**. **(J-L)** Co-expression of E5, E6, E7+hUBE3A+ mCD8RFP +p35 resulted in complete loss of Magi **(K)**. **(M-O)** Expression of mCD8RFP had no effect on Dlg **(N)**. **(P-R)** Co-expression of mCD8RFP +E5, E6, E7 had no effect on Dlg **(Q)**. **(S-U)** Co-expression of mCD8RFP+p35 did not affect Dlg level and localization **(T)**. **(V-X)** Co-expression of E5, E6, E7+hUBE3A+ mCD8RFP +p35 perturbed Dlg level and localization **(W)** but to lesser extent compared to Magi. Scale bar represents 100 μm.

HPV16 E5, E6, and E7 oncoproteins have been shown to target multiple cellular proteins, including PDZ domain proteins of polarity and junctional complexes, such as MAGI-1, hDlg1 and Scribble/Vartul, as well as the Retinoblastoma and p53 tumor suppressors [20, 23, 24, 26, 32, 69–71]. Since these targets have homologs in flies, we examined their levels and localization in tissues expressing HPV16 E5, E6, E7 alone, as well as in tissues co-expressing these oncogenes and hUBE3A in conjunction with p35. We compared them with the level and localization of these proteins in the control tissues that lacked any of the oncogenes. Immunolabeling of the third instar larval eye discs with antibodies to *Drosophila* Magi and Dlg revealed that co-expression of HPV16 oncogenes and hUBE3A led to the elimination of Magi from the epithelial cells (Fig 6J–6L). The PDZ domain protein, Dlg, was also absent from some regions, whilst other regions within the E5, E6, E7+ hUBE3A co-expression showed no effect on Dlg (Fig 6V–6X). Similar to our previous results, expression of HPV16 oncogenes, GMR-Gal4, or p35 alone did not cause any effects on Magi and Dlg levels and localization (Fig 6A–6I and 6M–6U). In contrast to PDZ domain proteins, *Drosophila* p53 was not targeted for degradation by HPV16 oncogenes. This is consistent with our previous studies indicating the inability of HPV18 E6 oncogene to eliminate *Drosophila* p53 due to differences in E6-binding domains between *Drosophila* and human p53 [49]. Altogether, these results are consistent with previous findings establishing Magi as a major degradation target of HPV16 and 18 E6 [24] and suggested that the mechanism of E6 targeting PDZ domain proteins of junctional and polarity complexes is conserved between human and *Drosophila*, both requiring the function of hUBE3A.

**Fig 7:**
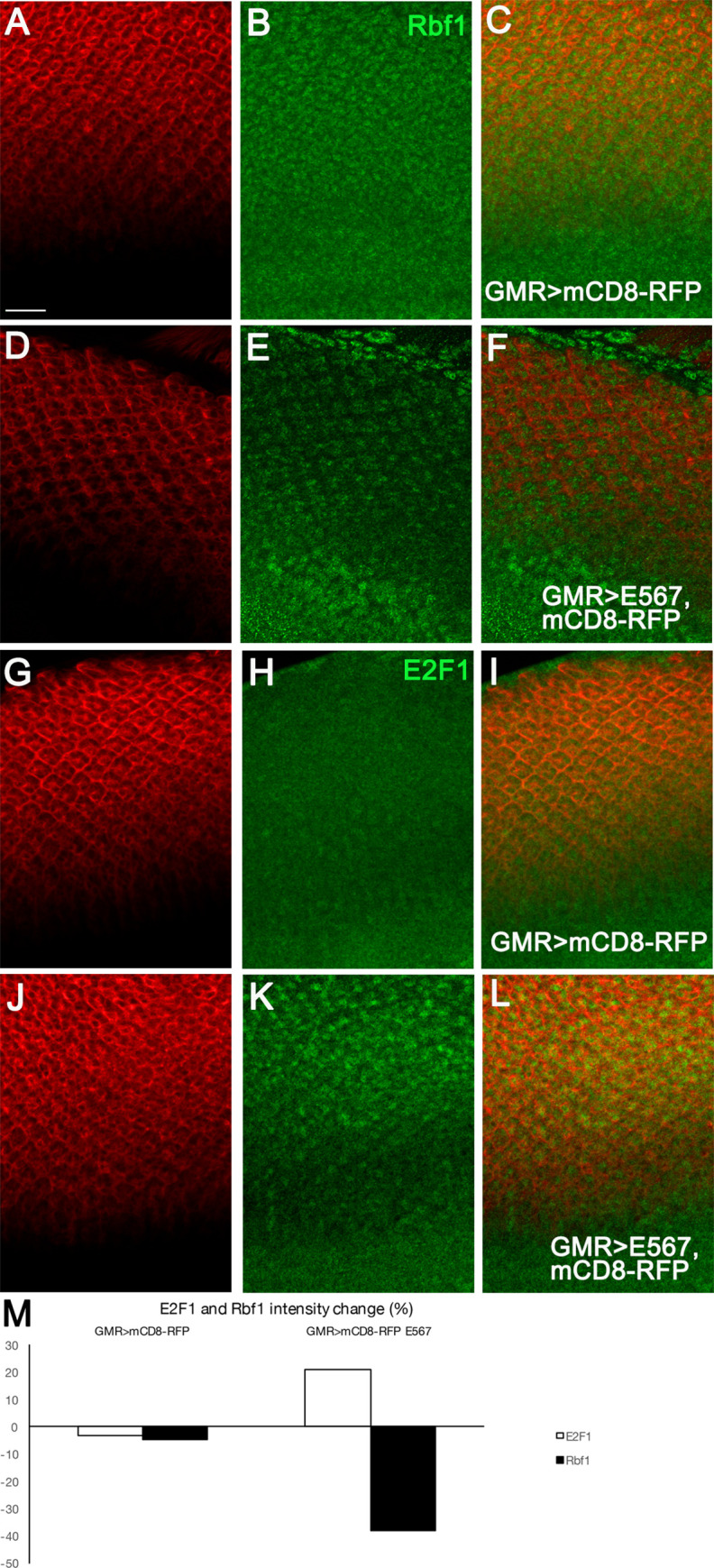
HPV16 E5, E6, E7 reduce the level of *Drosophila* Retinoblastoma protein Rbf1 resulting in an increase in E2F1. Transgenes were expressed in the third instar larval eye discs using GMR-Gal4. mCD8RFP was used to mark the transgenes expression region and tissues were immunolabeled for Rbf1 and its interacting protein, E2F1. **(A-C)** Expression of mCD8RFP had no effect on Rbf1, which was equally present in mCD8RFP-expressing and non-expressing regions **(B)**. **(D-F)** Co-expression of mCD8RFP +E5, E6, E7 led to reduction of Rbf1 **(E)**. **(G-I)** Expression of mCD8RFP had no effect on E2F1 and was present in low levels in the nuclei **(H)**. **(J-L)** Co-expression of mCD8RFP +E5, E6, E7 led to an increase in the level of E2F1 in nuclei of cells expressing the oncogenes **(K)**. **(M)** Quantification of the level of E2F1 and Rbf1 showed a significant reduction of the Rbf1 (38%, n = 6 eye discs) and a significant increase of E2F1 (21%, n = 7 eye discs) in eye discs expressing mCD8RFP +E5, E6, E7 compared to eye discs expressing mCD8RFP. Scale bar represents 100 μm.

In addition to E6 targets, we also examined the E7 target, pRb (Rbf1 in *Drosophila*). Immunolabeling with an antibody against Rbf1 revealed a significant reduction in the levels of Rbf1 in the nuclei of eye discs expressing HPV16 oncogenes, in comparison with control eye discs lacking the expression of these oncogenes (Fig 7A–7F and 7M). As Rbf1 binds to E2F1 and inhibits E2F1-dependent transcription during the G1 phase of the cell cycle [72–75], we examined the level and localization of *Drosophila* E2F1 in the presence of the E5, E6, E7 oncogenes and compared it with control eye disc that lacked the expression of these oncogenes. We found that eye discs expressing HPV16 oncogenes exhibited high levels of E2F1 in the nuclei of cells expressing the oncogenes, whilst those eye discs lacking the expression of the oncogenes showed very low level of E2F1 (Fig 7G–7M). These findings suggested that similar to E7-induced degradation of pRb in human cells, the *Drosophila* Rbf1 is a target of HPV16 oncogenes.

### HPV 16 E5, E6, and E7 oncoproteins induce the formation of long axon-like protrusions

**Fig 8:**
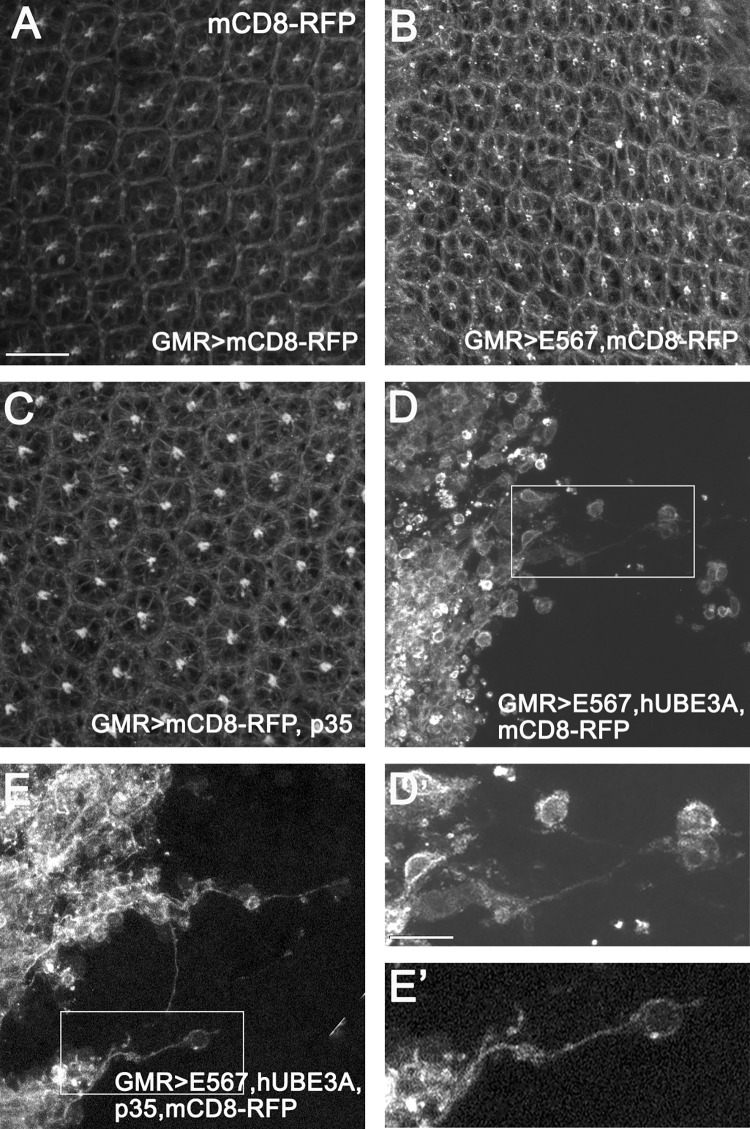
HPV16 E5, E6, and E7 oncoproteins induce the formation of long cellular protrusions. Transgenes were expressed in the epithelium of the eye tissues using GMR-Gal4. mCD8RFP was used to mark the cell membranes in the GMR-Gal4 expression region. All eyes are from 42 hours after pupation. **(A)** Pupal eye expressing mCD8RFP exhibited an array of hexagonal ommatidia arranged in stereotypical pattern. **(B)** Co-expression of mCD8RFP+E5, E6, E7 had no effect on the ommatidia formation and organization. **(C)** Co-expression of mCD8RFP+E5, E6, E7+p35 did not perturb ommatidia development and arrangement. Co-expression of E5, E6, E7+hUBE3A+ mCD8RFP **(D, D’)** or co-expression of E5, E6, E7+hUBE3A+ mCD8RFP+p35 **(E, E’)** both resulted in loss of ommatidia differentiation. Pupal eye showed clusters of cells with rounded cell morphology. A subset of these cells were malignant and found at distant sites from the cluster **(D, E)**. Some of these cells extended long cellular protrusions originating from opposing regions of the cell that linked the metastatic cells to each other or to the cells within the cluster (D’ and E’ magnified from boxes in D and E). Insets are digitally magnified 200%. Scale bar in A indicates 100 μm and in insets represents 50 μm.

**Fig 9:**
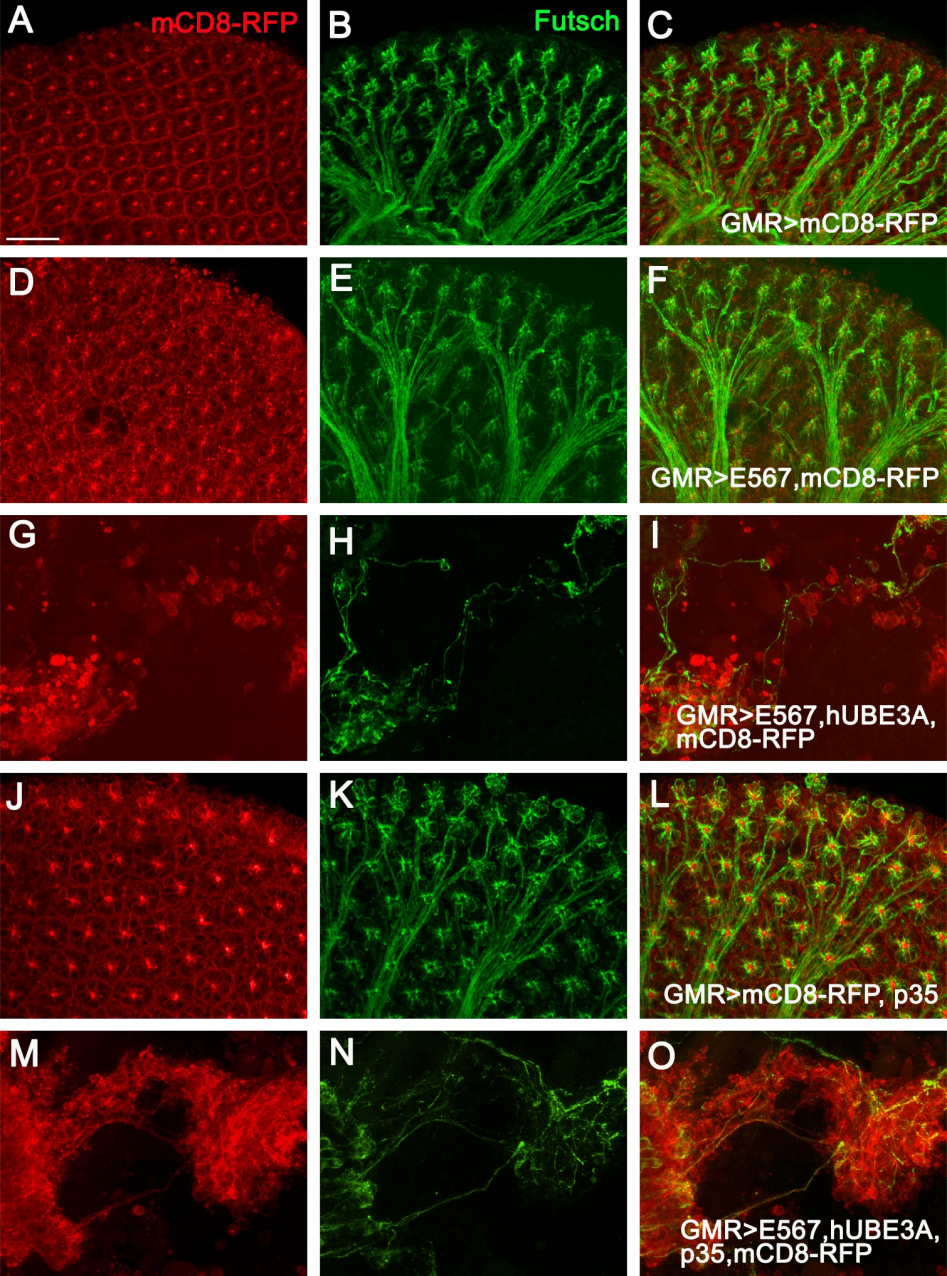
HPV16 oncogenes in cooperation with hUBE3A induce the transformation of epithelial cells to neuron-like cells. Transgenes were expressed in the epithelium of the eye tissues using GMR-Gal4. mCD8RFP was used to mark the cell membranes in the GMR-Gal4 expression region. All eyes are from 42 hours after pupation and immunolabeled for axonal marker, Futsch. **(A-C)** Pupal eye expressing mCD8RFP exhibited a stereotype pattern of hexagonal array of ommatidia **(A)** from which only the photoreceptor neurons of each ommatidia project axons that fasciculate together and project as a single bundle towards the optic lobes of the brain **(B)**. Co-expression of mCD8RFP+E5, E6, E7 **(D-F)** or co-expression of mCD8RFP+E5, E6, E7+p35 **(J-L)** had no effect on ommatidia differentiation and photoreceptor axonal projections. Pupal eyes co-expressing E5, E6, E7+hUBE3A+ mCD8RFP **(G-I)** or E5, E6, E7+hUBE3A+ mCD8RFP+p35 **(M-O)** showed no differentiated ommatidia but instead clusters of cells **(G, M)** with rounded cell morphology and cellular protrusions that expressed the axonal marker Futsch **(H, N)**. The axon-like protrusions were developed by both the malignant cells and cells within the primary cluster **(I, O)**. Scale bar represents 100 μm.

HPV-induced cancer cells develop malignant properties in advanced stages of cancer, leaving the primary tumor and migrating to distant sites. Loss of cell polarity mediated by HPV oncogenes facilitates the epithelial to mesenchymal transition (EMT) and malignancy [68, 76]. As co-expression of HPV16 E5, E6, E7+hUBE3A in larval eye disc had perturbed cell polarity, it prompted us to investigate whether these depolarized cells had acquired invasive phenotypes later during the pupal stage of development. We discovered that while pupal eyes expressing either GMR-Gal4, p35, or (E5, E6, E7) alone exhibited an intact eye with a stereotypical pattern of hexagonal ommatidia (Fig 8A–8C), eyes expressing either HPV16 E5, E6, E7+hUBE3A or HPV16 E5, E6, E7+hUBE3A+p35 did not contain any ommatidia, but instead had clusters of cells that had acquired rounded cell morphology with a subset of cells emanating cellular protrusions that were originated from two different regions of the cells. These cells, which were found to be malignant and in distant sites from the cell cluster, retained their attachment with either other migratory cells or cells within the primary cluster through these long protrusions (Fig 8D–8E’). These observations led us to investigate whether these protrusions were axonal and a result of cell transformation. Immunolabeling for Futsch, the *Drosophila* homolog of human MAP1B, a neuron-specific microtubule-associated protein that is widely used to visualize neuronal morphology and axonal projections [77] revealed that these protrusions expressed Futsch indicating that they were axon-like (Fig 9G–9I and 9M-9O). Furthermore, we discovered that not only the malignant cells but the cells within the primary cluster developed these protrusions that held the depolarized cells together. These results were in contrast to the eyes expressing either GMR-Gal4, p35, or (E5, E6, E7) alone. In these intact eyes with proper cell differentiation and a stereotypical pattern of a hexagonal array of ommatidia, only the photoreceptor neurons of each ommatidium extend axons that fasciculate together and project as a single bundle towards the optic lobes of the brain. The other cells of the ommatidia are non-neuronal and hence do not express Futsch (Fig 9A–9F and 9J–9L). These results are novel and altogether suggested that HPV16 oncogenes in cooperation with hUBE3A induce the transformation of epithelial cells to neuron-like cells, a process that requires gaining an axonal gene expression program.

## Discussion

Here in this study, we developed the first *Drosophila* model of HPV16-induced cancer through the expression of its main oncogenes, E5, E6, E7 in conjunction with human UBE3A. We found that several dysregulative actions of these oncogenes in human cells also occur in *Drosophila* epithelial cells, including loss of cell adhesion and polarity, degradation of PDZ domain proteins and Rbf, disruption of cell differentiation, and cell transformation, as well as malignancy. These findings suggest that the *Drosophila* model of HPV16-induced cancer can serve as an excellent complementary model to the existing human cell culture system and transgenic mice models, thanks to its advantages in large-scale *in vivo* genetic and high-throughput drug and compound screening. We further showed that unlike HPV18E6, which caused pupal eye ommatidial abnormalities [49], expression of HPV16 oncogenes in conjunction with hUBE3A cause more severe cellular alterations. These alterations result in lack of eye structures but instead promote formation of cell clusters with migratory and malignant behaviors. These findings suggest that E6 cooperates with E7, and perhaps with E5 and is consistent with previous studies demonstrating the necessity for cooperative action of E6 and E7 to induce cellular transformation [78, 79]. Additionally, HPV16 oncogenes E5, E6, E7 plus hUBE3A caused pupal lethality. This pupal death is not due to malignancy as we did not find any malignant cells outside of the eye region. Hence, pupal lethality is likely due to transforming activity of the oncogenes in cells outside of the eye region as GMR-Gal4 has been shown to have a broad expression profile rather than an eye-specific pattern of expression [80]. Additionally, our results indicate that, similar to our previous finding for HPV18 E6, the HPV16 E6 oncogene lacks the ability to disrupt cellular homeostasis without the assistance of hUBE3A [49]. Although the mechanism of proteasomal-mediated degradation of cellular proteins is conserved between humans and fruit flies, however, the *Drosophila* UBE3A does not possess the E6-binding motif, hindering it from interacting with this oncogene [81]. Additionally, it suggests that E6 plays a crucial role in HPV-induced cancer, as E5 and E7, despite E7’s ability to eliminate Rbf1, lacked the ability to cause cellular transformation in the absence of hUBE3A interaction with E6. This is consistent with previous studies indicating an essential role for targeted degradation of PDZ domain proteins of junctional and polarity complexes by E6 in tumorigenesis [29, 30] as well as the necessity for cooperative action of E6 and E7 to induce cellular transformation [78, 79]. Furthermore, the lack of *Drosophila* p53 degradation in our model suggests that similar to HPV18 [49], HPV16-induced disruption of cellular homeostasis, cancer development, and malignancy is independent of the p53 degradation. This result is in agreement with previous findings [29, 30, 82] and suggests that our *Drosophila* model of HPV16-induced malignancy is ideal for understanding p53-independent mechanisms of cancer mediated by HPV16 oncogenes.

One interesting finding in our study was heterogeneity in response to HPV16 oncogenes. We found that not all cells in the region of larval eye disc expressing HPV oncogenes in conjunction with hUBE3A, responded equally. While some cells within the population had lost cell adhesion and undergone programmed cell death, others had not. The remaining cells managed to progress through the next developmental stages. However, these cells lacked the ability to differentiate to any cell types of the eye and instead transformed into neuron-like cells extending protrusions to migrate while still connected to their peers. The apoptotic cell death phenotype and lack of cell proliferation despite E7-induced loss of Retinoblastoma could be due to action of p53 as HPV E6 does not have the ability to eliminate *Drosophila* p53 due to differences in E6-binding domains between *Drosophila* and human p53 [49]. This is consistent with previous studies showing that HPV16 and 18 E7 cause apoptotic cell death in the presence of an intact p53 and that p53 elimination is necessary for E7-mediated cellular transformation and tumor formation [83–88]. The differential response among cells could lie in the effect of oncogenes being dosage-dependent and correlates with the degree of oncogene expression. We have reported similar observations for a HPV18 E6-induced effect, in which only a subset of the cell population co-expressing E6 and activated Ras were transformed to cancer-like cells and those were the cells that expressed higher levels of E6 oncogene [49]. Single-cell clone analysis of HPV18 E6 and E7 positive human esophageal cancer cells also showed that the intra-tumor heterogeneity is due to variation in the level of E6 and E7 expression, with cells expressing higher levels exhibiting cell proliferation and invasive behavior [89]. Hence, the *Drosophila* model could be a valuable *in vivo* system for understanding the mechanisms underlying the intra-tumor genetic heterogeneity, which is currently a great problem in the treatment of cancers including HPV-associated cancers [90, 91].

One of the important hallmarks of cancer including HPV-induced cancer is malignancy. We and other studies have shown that E6 or E7 alone lack the ability to cause malignancy [37, 49, 78] and that tumorigenesis requires the cooperative action of both oncogenes. In agreement with these results, we found that the presence of HPV16 E5, E6, E7 oncoproteins in conjunction with hUBE3A is necessary for cell transformation and development of malignant properties. Among these properties was a novel characteristic that was not previously reported for HPV+ cancer cells: the extension of axon-like structures by migrating cells that were leaving the transformed cell cluster, as well as by a subset of cells within the cluster. Our further investigation revealed that HPV16 oncogenes induced cellular transformation to neuron-like cells. Gaining a neuronal gene program has been previously reported for metastatic cancer cells of other types of tumors and shown to contribute to and facilitate their metastatic behavior [92, 93]. However, the mechanisms underlying this epithelial to neuronal transition including the change in molecular program, the formation of protrusions, and the connectivity of cancer cells through these protrusions are not understood. We believe that our *in vivo* whole-animal model would be extremely useful in understanding these mechanisms, due to its advantages in whole-genome genetic and high-throughput compound screening. These screenings can lead to identification of signaling pathways and genes that play important roles in epithelial to neuronal transition, as well as aiding in the discovery of inhibitory compounds that would provide clinical benefit.

Furthermore, our study reveals that several key cellular targets of HPV16 oncogenes are well-conserved in *Drosophila* and are equally targeted for degradation. Although E6-mediated elimination of PDZ domain proteins requires the human UBE3A, the ubiquitination and subsequent proteasomal degradation of *Drosophila* Retinoblastoma by E7 did not require the human cullin 2 *ubiquitin ligase complex [94]*, suggesting that the *Drosophila* counterpart represents a conserved binding activity of E7. Indeed the subunit within the Cullin 2 ubiquitin ligase complex with which E7 interacts, Elongin C, is well conserved in *Drosophila* with more than 95% sequence homology [95, 96]. These results suggest that HPV16 oncogene-mediated elimination of cellular targets involves an evolutionarily-conserved mechanism. Similar conserved mechanisms of virus-host interactions between fruit flies and humans have been demonstrated for other viruses including HIV, ZIKV, Epstein-Barr, VSV, and SARS-CoV [45, 97–101]. Therefore, the findings from this study, places the *Drosophila* model of HPV16 oncogenes-induced malignancy in a unique position for understanding the disease mechanism and discovery of novel molecular targets for HPV-associated cancer treatments.

## Supporting information

S1 Fig Combined expression of HPV16 E5, E6, E7 and hUBE3A has no effect on the mitotic cell division during larval eye disc patterning and development. Transgenes were expressed in the epithelium of the third instar larval eye tissues using GMR-Gal4. mCD8RFP was used to mark the cell membranes in the GMR-Gal4 expression region. Immunolabeling for mitotic cell marker phosphohistone 3 (pH3) revealed that co-expression of mCD8RFP+E5, E6, E7 **(A-C)** or E5, E6, E7+hUBE3A+ mCD8RFP **(D-F)** had no effect on the level of mitotic cell divisions as it exhibited the same result as the control **(G)** in which only the GMR-Gal4 was expressed. Scale bar represents 100 μm.
(TIF)
