# Supplementary figures and images for: A Drosophila model of HPV16-induced cancer reveals conserved disease mechanism
Source: PLoS One. 2022 Dec 12;17(12):e0278058. doi: 10.1371/journal.pone.0278058 (PMC9744332; doi:10.1371/journal.pone.0278058)

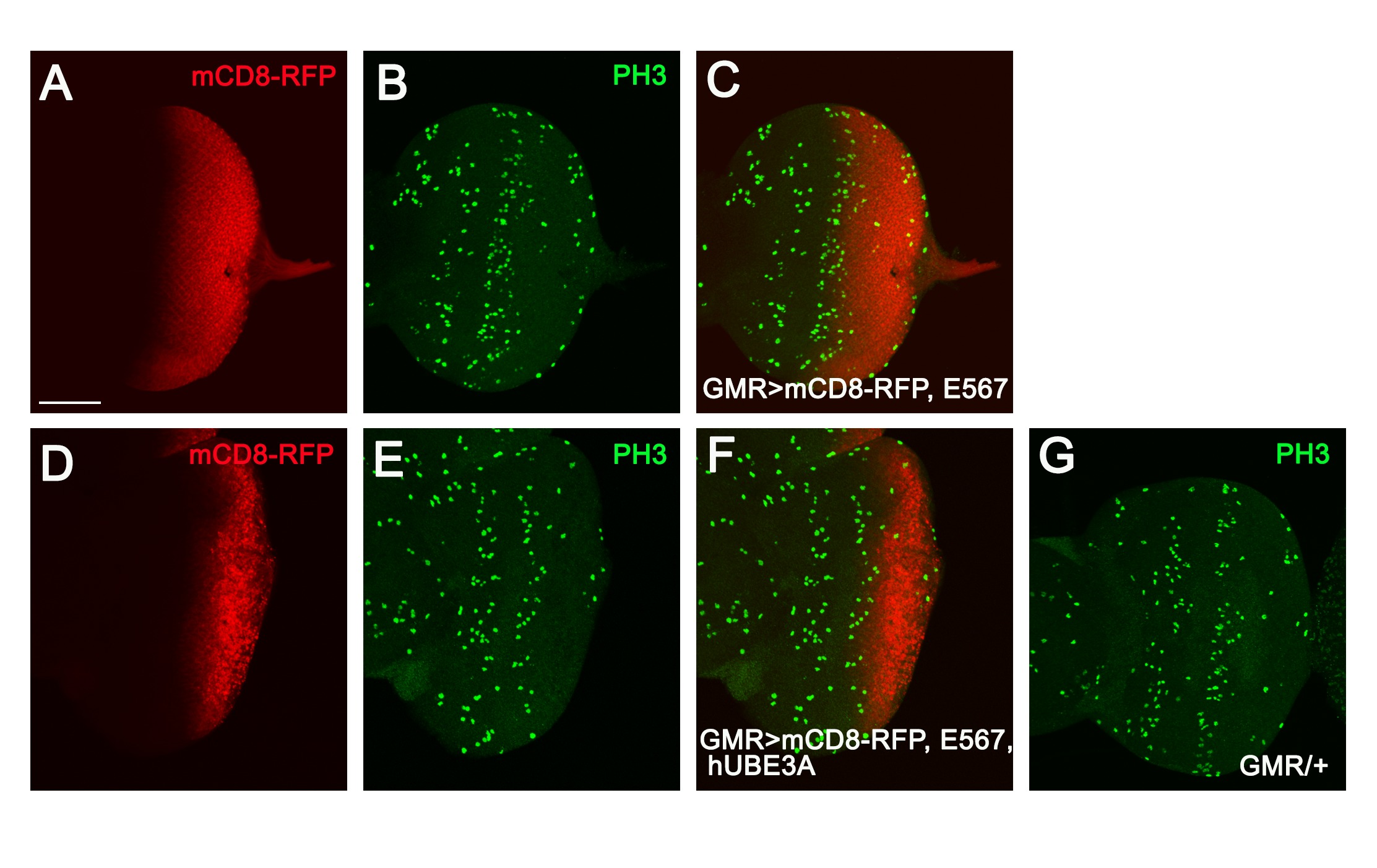

Supplement: S1 Fig — Transgenes were expressed in the epithelium of the third instar larval eye tissues using GMR-Gal4. mCD8RFP was used to mark the cell membranes in the GMR-Gal4 expression region. Immunolabeling for mitotic cell marker phosphohistone 3 (pH3) revealed that co-expression of mCD8RFP+E5, E6, E7 (A-C) or E5, E6, E7+hUBE3A+ mCD8RFP (D-F) had no effect on the level of mitotic cell divisions as it exhibited the same result as the control (G) in which only the GMR-Gal4 was expressed. Scale bar represents 100 μm. (TIF) [file pone.0278058.s001.tif]
